# Case of Strangurated Inguinal Hernia, Successfully Treated

**Published:** 1859-03

**Authors:** H. De La Cossitt

**Affiliations:** Greenville, Mercer Co., Pa.


ART. IV.—*Case of Strangurated Inguinal Hernia, Successfully Treated.* By H. De La Cossitt, M. D., Greenville, Mercer Co., Pa.

On the evening of the 29th of November, 1858, John Carmical, of Pymenturning, Mercer county, Pa., aged 35, when returning home from one of his neighbors, discovered a hardness and fullness of the left side of the scrotum, which caused pain in his bowels, and vomiting.

At 12 o'clock, P. M., I was called to visit him, but did not go; the messenger informed me he had taken cold, and his privates were swollen, and he was suffering great pain. I sent, then, some medicine.

On the second day after, I visited him. On examination, found a case of hernia, the scrotum largely distended, hard and purple.

I bled, gave tobacco injections, warm bath, and used every reasonable means, but could not reduce the protrusion.

The case progressed until the morning of the fifth day, when he agreed to submit to an operation.

After removing the hair from the parts, I placed him on a table, with his shoulders somewhat elevated, and his legs hanging over the end of the table, placing his feet on stools, and one assistant on each side of him. I then made an incision, about two inches, through the integuments, dissected to the sac, and found a portion of the omentum; then enlarged the opening to four inches, when blood and serum, to the amount of a gill, discharged; then extirpated the omentum; supposed it would weigh six ounces. It was black and fetid; the intestine extended down five inches, also black, (his bowels tumid and tender. After cleansing the parts with a sponge and warm water, I introduced my left index finger to the stricture, and followed it with the probe-pointed bistoury, which I passed under the stricture, enlarged the opening, then returned the intestine, brought the wound together with sutures, adhesive straps, and a compress, and secured them with a roller; gave castor oil and turpentine for cathartic. His bowels opened on the second day; his pulse was 120, attended with sweat and hiccup.

Quinine, ammonia, wine, spirits of nitre, and turpentine, were ordered to be given, as his situation demanded.

All passed off well for eight days, when I visited him, and found the excrements passing out at the wound, which continued for fifteen days. During that time there was a natural stool every two or three days; the same dressing was continued, being removed and cleaned once every day. The wound closed on the 36th day from the operation; the natural passage was good every day. I discharged him from further attention. He is now convalescent. Such cases should not be delayed over the second day; an operation should be resorted to when the circumstances demand.

This is the fifth case that I have operated upon for hernia; three recovered. We, in a country practice, are not permitted to operate until often too late to save the patient. We are taught in this case never to despair, no matter how gloomy or desperate the case may be. Nature is the great architect, requiring from man his assisting scalpel, which he must use with discrimination and dexterity.

